# Delphi consensus on strategies in the management of opioid-induced constipation in cancer patients

**DOI:** 10.1186/s12904-020-00693-z

**Published:** 2021-01-02

**Authors:** Regina Gironés Sarrió, Agnès Calsina-Berna, Adoración Gozalvo García, José Miguel Esparza-Miñana, Esther Falcó Ferrer, Working Group ActEIO Project, Rosa María Villatoro Roldan, Jaime Alcober Pérez, Vicent Alcolea Fuster, José Huidobro Ubierna, Begoña Campos Balea, Fernando García Urra, Antonio Gabriel Amengual Vich, Olga Linares Alemparte, Sandra Villamil Montufar, Miguel Ángel Núñez Viejo, Virginia Aurora Ochagavia Galilea, Esther García Asencio, Ana Ruiz Alonso, Alfredo Paredes Lario, María del Carmen Francisco López, Visitación Sorolla Gutiérrez, Núria Calvo Vergés, Natalia Iriarte Gay de Montella, Marta Andrés Escapa, Juan David Cárdenas, María Escarlata López Ramírez, Laura Visa, Julio César Melón Pérez, Juan Luis Martí Ciriquian, Raquel Marsé Fabregat, Joaquina Martínez Galán, Isabel Chirivella González, Margarita Álvaro Pardo, Luis Enrique Chara Velarde, María Isabel Villanego Beltrán, Paula Peleteiro Higuero, María Fernández Abad, Consuelo Rodríguez Rodríguez, José Lorenzo Gómez-Aldaraví, Cristina Mateo, Javier Martínez Trufero, María Sereno Moyano, Sandra Morales Pérez, Roberto A. Pazo Cid, Deborah Moreno Alonso, Salvador Garcerá Juan, Fernando Henao Carrasco, José Agustín González Romero, Alejandro Falcón González, Carlos López López, María Herrera Abián, Josep Porta-Sales

**Affiliations:** 1 Medical Oncology Department, Hospital Universitari i Politècnic La Fe, Avinguda Fernando Abril Martorell, 106, 46026 Valencia, Spain; 2 Department of palliative care, Institut Català d’Oncologia-Badalona (ICO-Badalona), Badalona, Spain; 3 Responsable de la Unidad de Hospitalización Domiciliaria, Hospital Universitario Doctor Peset, Valencia, Spain; 4 Escuela de Doctorado, Universidad Católica de Valencia San Vicente Mártir. Hospital de Manises, Valencia, Spain; 5 Department of Anaesthesiology, Critical Care and Pain Treatment. Research Group in Perioperative Medicine, Hospital Universitario y Politécnico La Fe, Valencia, Spain; 6 Medical Oncology Department. Hospital Son Llàtzer, Palma, Mallorca Spain; 7 Department of palliative care, Institut Català d’Oncologia-Girona (ICO-Girona), Girona, Spain

**Keywords:** Cancer, Constipation, Opioid, Laxative, Pain

## Abstract

**Background:**

Opioid-induced constipation (OIC) is a frequent and bothersome adverse event related with opioid therapy in cancer patients. Despite the high prevalence, medical management of OIC is often uncertain. The current project aimed to investigate expert opinion on OIC management and provide practical recommendations to improve the clinical approach of OIC in cancer patient.

**Methods:**

A modified Delphi method was conducted involving 46 different physicians experts in OIC. Using a structured questionnaire of 67 items this project intended to seek consensus on aspects related to diagnosis, treatment, and quality of life of cancer patients suffering with OIC.

**Results:**

After two rounds, a consensus was reached in 91% of the items proposed, all in agreement. Agreement was obtained on OIC definition (95.7%). Objective and patient-reported outcomes included in that definition should be assessed routinely in clinical practice. Responsive to symptom changes and easy-to-use assessment tools were recommended (87.2%). Successful diagnosis of OIC requires increase clinicians awareness of OIC and proactivity to discuss symptoms with their patients (100%). Successful management of OIC requires individualization of the treatment (100%), regular revaluation once is established, and keeping it for the duration of opioid treatment (91.5%). Oral Peripherally Acting μ-Opioid Receptor Agonists (PAMORAs), were considered good alternatives for the treatment of OIC in cancer patients (97.9%). This drugs and laxatives can be co-prescribed if OIC coexist with functional constipation.

**Conclusions:**

The panelists, based on their expert clinical practice, presented a set of recommendations for the management of OIC in cancer patients.

**Supplementary Information:**

The online version contains supplementary material available at 10.1186/s12904-020-00693-z.

## Background

Pain prevalence in patients with cancer ranges from 39.3% after curative treatment, 55.0% during anticancer treatment, and 66.4–80% in advanced disease [1]. Because of this high prevalence, it is a priority to establish adequate control of pain in these patients [2]. Opioids are one of the main treatment options in patients with severe pain [3, 4]. Despite their great efficacy, opioids are associated with several adverse events including constipation, nausea, vomiting, dry mouth, oesophageal reflux or abdominal discomfort. Some of them, such as nausea and vomiting, disappear over time, but OIC can occur from the start of opioid administration and be present throughout opioid treatment [5, 6].

OIC is a frequent and distressing disorder in cancer patients, with a significant impact on their quality of life [7–10]. OIC occurs in 51–87% of patients receiving opioids for cancer [11]. Although OIC is one of the most common causes of constipation in cancer patients, there are many other factors that can contribute to the development of constipation or exacerbate OIC symptoms [12–14]. A differential diagnosis of OIC is necessary to identify underlying causes of constipation, and to provide specific treatment [11–13]. Functional constipation and OIC have similar presentation, but their physiopathology is different. They have been well-defined in Rome IV criteria publication [12].

Traditionally, the treatment of OIC has been based on hygienic-dietary measures and the use of laxatives. However, it has been proven that these measures have not been sufficiently effective in a high percentage of cases [15–17]. This may due to the fact that they do not act on the underlying mechanism of the OIC and the recommendations are based mainly on data obtained from patients with functional constipation.

In the last decade, major contributions have been made to elucidate the complexity of OIC physiopathology and to develop new targeted drugs. These new agents, in contrast to conventional treatment, are specific for OIC and target the underlying mechanism of OIC. The PAMORAs, preferentially block μ-opioid receptors in the periphery and do not interfere with the analgesic effects of opioids in the central nervous system [18, 19].

Despite all this progress and the publishing of some clinical guidelines in the management of OIC [13, 20], there is still no comprehensive clinical guideline that approach the complexity of OIC in cancer patients in real world clinical practice. In order to develop practical and valuable tools that help us to improve the clinical management of OIC in cancer patients, we have performed a Delphi consensus to gather the opinion of different Spanish physicians, experts with extensive knowledge and experience on opioid use and OIC.

## Methods

### Study design

This study was performed according to a modified Delphi method. The goal was to reach a consensus on key aspects of the medical management of OIC in cancer patients, based on evidence and expert’s clinical experience [21–23]. The Delphi was carried out in 3 stages: 1) a face-to-face meeting with a scientific committee to raise the main topics; 2) two successive rounds of online surveys to gather the opinion of a panel of experts; and 3) result analysis and conclusions discussion by the scientific committee in a face-to-face meeting. Blind voting was carried out, and to minimize the band-wagon effect in the meetings, it was ensured that all the participants spoke the same amount of time.

### Participants

A group of 46 health care providers (HCP) (48% medical oncologists, 33% from palliative care, 17% clinical oncologists, and 2% from pain unit), with more than 3 years of clinical experience in cancer pain treatment with opioids and OIC, were involved in this consensus. Experts were representative from all regions of Spain.

### Questionnaire

A scientific committee, in a face-to-face start-up meeting, debated the main diagnostic and treatment strategies of OIC in cancer patients, as well as those aspects related to their quality of life. As a result of the debate, a questionnaire of 67 items was made in the following subjects: 1) Diagnosis of OIC (21 items); 2) Treatment of OIC (38 items); and 3) Quality of life of cancer patients with OIC (8 items).

A nine point ordinal Likert-type scale was used for the purpose of answer to the items, as stated by the model developed by UCLA-RAND Corporation (minimum 1, full disagreement; and maximum 9, full agreement) [21]. The scale was structured in three groups, according to the level of agreement-disagreement of the item: from 1 to 3, it means a rejection or disagreement; from 4 to 6, no agreement or disagreement; and from 7 to 9, interpreted as expression of agreement or support.

### The Delphi rounds and consensus meeting

Two rounds was conducted to answer the Delphi questionnaire, carried out between May and July 2018. All the respondents were blinded for each other opinions. During the 1st round, expert panellists answered the on-line Delphi questionnaire, and had the option to add their opinion and comments about it. Then the results were assessed and presented through bar graphs, in order to synthesize the opinions from each participant. With this information, in the 2nd round, the panellists were able to respond again to those questions that did not reach consensus. The results of this second round were tabulated and presented descriptively in a face-to-face meeting of the scientific committee. It was carried out on September 2018, with the objective to debate and analyse the final results.

### Analysis and interpretation of results from the questionnaires

The results of the questionnaire was evaluated through the score and the level of consensus reached. The same criteria for each of the items were followed. The consensus criteria was established when two-thirds or more of the answers scored within the 3-point range (1–3 or 7–9) containing the median. The median value of the score established the type of consensus achieved on each item. Agreement when the median of the score was ≥7, disagreement in the cases that the median was ≤3. The items were considered uncertain when the median of the score was located between the 4–6 range.

## Results

### Delphi consensus

**Fig. 1:**
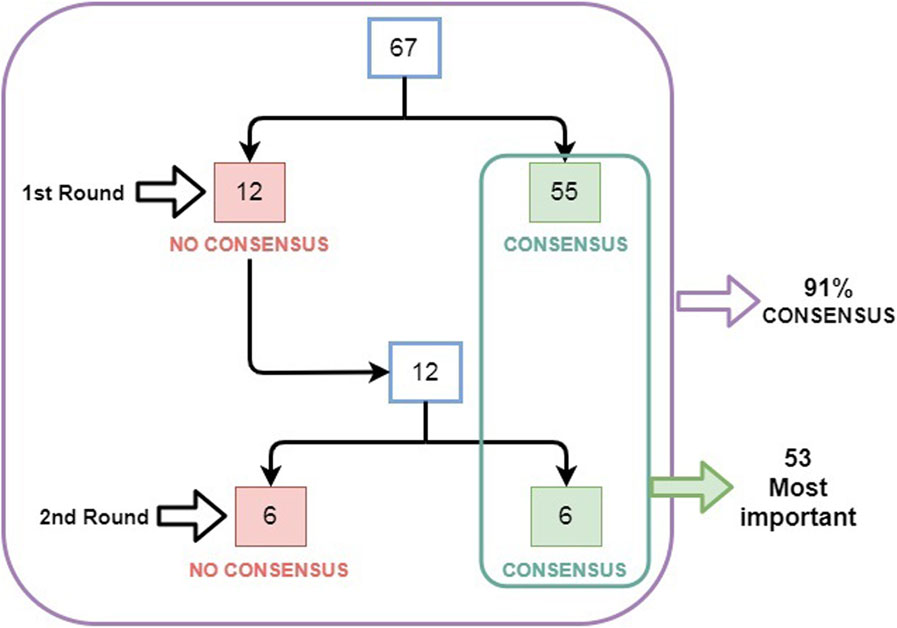
Main results of the Delphi consensus

All 46 experts consulted completed the two rounds of the Delphi consensus without proposing new items. In round 1, consensus was reached on 55 of 67 items. Twelve items were returned for reconsideration in round 2 and consensus was reached in 6 items. After two rounds, a consensus was reached in 61 items (91%); all in agreement. The remaining 6 items (9%) did not show agreement or disagreement. The most important items (53 of 67) have been selected to be included in this publication. Figure 1 depicts the results of the two rounds and Tables 1, 2, and 3 show the global results for the 53 selected items.

**Table 1:** Agreement achieved by the experts after the two rounds (Topic 1)

	Median (IQR)	Agreement (%)
**Topic 1. Diagnosis of OIC**		
1. OIC is defined as: “a change in previous bowel habits when opioid treatment is initiated or modified that is characterized by any of the following conditions: decreased frequency of spontaneous bowel movements, straining of stools, feeling of incomplete bowel movements, harder stools, perception of patient involvement in relation to bowel habits.	8 (1)	95.75
2. The use in clinical practice of the ROME IV criteria may contribute to improve the differential diagnosis of OIC in cancer patients.	8 (2)	82.97
3. The use of the Bowel Function Index (BFI) questionnaire in daily clinical practice would facilitate the diagnosis of OIC.	8 (2)	74.47
4. The comprehensive approach to OIC is different in oncology patients from that of non-oncology patients.	8 (2)	90.00
5. OIC is a common problem among cancer patients in routine clinical practice	8 (1)	87.24
6. There are a large number of patients with cancer affected by OIC in which a correct diagnosis of this condition is not made.	8 (2)	85,1
7. For an effective approach to OIC, it must be remembered, both at the time of prescription and throughout treatment, that opioids can cause constipation.	9 (1)	100.00
8. It is recommended to educate patients on OIC (causes, symptoms, management, etc.) when receiving opioid treatment.	9 (1)	97.88
9. It is recommended that healthcare professionals proactively ask patients under opioid treatment about the symptoms of OIC in order to improve this condition.	9 (1)	100.00
10. Occasionally, patients only report symptoms of OIC to the physician when these are severe.	8 (2)	85.11
11. At the time of opioid treatment it is important to know the presence of previous functional constipation (constipation caused by causes other than opioids).	8 (1)	93.62
12. When OIC coexists with other causes of constipation, the symptoms of OIC are exacerbated.	8 (1)	89.37
13. For the effective diagnosis of OIC, a complete patient clinical history (including previous history, pharmacological treatments, metabolic alterations...) is needed to help differentiate OIC from functional constipation.	9 (1)	95.75
14. For the effective diagnosis of OIC, it is essential to evaluate the temporal relationship between the onset of opioid use and the development of OIC symptoms.	8 (2)	89.36
15. For the effective diagnosis of OIC in oncological patients, a physical examination is essential to rule out possible organic problems that could be the origin of the symptoms, such as intestinal obstruction.	8 (1)	89.37
16. At the time of diagnosis of OIC, it is essential to evaluate the presence of faecal impaction.	8 (2)	78.72
17. In routine clinical practice, requests for additional tests are necessary only if other causes of constipation are suspected.	8 (1)	80.84

*IQR* interquartile range, *OIC* opioid-induced constipation

**Table 2:** Agreement achieved by the experts after the two rounds (Topic 2)

	Median (IQR)	Agreement (%)
**Topic 2. Treatment of OIC**		
18. Prevention and early treatment (when symptoms are still mild) are recommended to anticipate the development of OIC.	9 (1)	97.87
19. Management of OIC becomes more complicated if treatment is started when OIC is already established and symptoms are more severe.	9 (1)	95.74
20. After scheduling an OIC treatment, frequent reassessment is recommended to optimize the treatment.	8 (1)	97.87
21. Effective OIC management involves maintaining OIC treatment while using opioid treatment.	9 (2)	91,48
22. For a better approach to OIC, it would be recommended to have simple questionnaires with criteria to assess the effectiveness of treatment for OIC.	9 (1)	93.62
23. For the treatment of OIC in oncological patients with polypharmacy, it is recommended to use drugs with comfortable administration.	9 (1)	97.87
24. Posology simplification of single daily dose oral treatments contributes to improve the patient’s satisfaction with the treatment.	9 (1)	97.87
25. High patient satisfaction with the prescribed treatment contributes to improving the efficacy of OIC treatment.	9 (1)	91.49
26. Good therapeutic adherence contributes to improving the efficacy of OIC treatment.	9 (1)	97.87
27. A good therapeutic strategy for OIC involves an individualization of the treatment, adapting it to the needs of each patient.	9 (1)	100.00
28. For a comprehensive approach it is necessary to treat both OIC and functional constipation, if both coexist.	8 (1)	91.49
29. Because of its pathophysiology, effective management of OIC requires a specific treatment that acts on the underlying cause.	8 (1)	95.75
30. Although hygienic-dietary recommendations are necessary for the treatment of OIC, they are not sufficiently effective.	8 (2)	91.48
31. Laxatives are often not effective for the treatment of OIC in cancer patients.	8 (2)	78.73
32. Oral PAMORA, such as naloxegol, are a good therapeutic alternatives in the treatment of OIC in cancer patients.	9 (1)	97.87
33. The use of enemas for the treatment of OIC in cancer patients should be used occasionally.	7 (2)	74.47
34. It is recommended to maintain the laxative dose when starting opioid treatment in cancer patients with functional constipation.	8 (2)	78.72
35. It is recommended to assess the efficacy of OIC treatment as soon as possible, especially during the first week after the start of treatment.	8 (2)	91.50
36. According to clinical practice, if the laxative has not met the therapeutic objectives for OIC treatment, it is recommended to take oral PAMORA (e.g. naloxegol, etc) while maintaining the dosage of the laxative as prescribed.	8 (2)	80.00
37. According to clinical practice in oncological patients with OIC, if the administration of oral PAMORA (e.g. naloxegol, etc.) in monotherapy has not fulfilled the therapeutic objective, the coexistence of other factors in the origin of constipation should be considered.	8 (2)	87.23
38. According to clinical practice in oncological patients with OIC, if the administration of oral PAMORA (e.g. naloxegol, etc.) in monotherapy has not fulfilled the therapeutic objective, adjuvant treatment with oral PAMORA and osmotic laxative should be recommended.	7 (2)	72.33
39. According to clinical practice in oncological patients with OIC and functional constipation, if adjuvant treatment with oral PAMORA and laxative has not met the therapeutic objective, occasional use of enemas may be considered.	8 (1)	78.72
40. According to clinical practice in oncological patients with OIC and functional constipation, if adjuvant treatment with oral PAMORA and laxative has not met the therapeutic objective, change between opioids may be considered.	8 (2)	72.34
41. Greater benefits are achieved from treatment with oral PAMORA (e.g. naloxegol, etc.) when started early.	8 (2)	93.62
42. If patients have severe symptoms of OIC with fecal impaction, it is recommended that a disimpaction be performed before starting treatment with oral PAMORA (e.g. naloxegol, etc.).	8 (1)	78.72
43. Poor OIC control can increase the number of medical visits.	9 (1)	93.62
44. Poor OIC control can increase the number of emergency visits.	9 (1)	91.49
45. Poor OIC control can lead to increased healthcare costs.	8 (1)	97.87

*IQR* interquartile range, *OIC* opioid-induced constipation, *PAMORA* peripherally acting mu-opioid receptor antagonists

**Table 3:** Agreement achieved by the experts after the two rounds (Topic 3)

	Median (IQR)	Agreement (%)
**Topic 3. Quality of life of cancer patients with OIC**		
46. OIC negatively affects the quality of life of patients with cancer.	9 (1)	97.88
47. Poor OIC control can negatively affect adherence to pain management, affecting the quality of life of patients with cancer.	9 (1)	95.74
48. The simplification of the dosage of oral treatments in a single daily dose contributes to improve the quality of life of patients with OIC.	9 (1)	93.61
49. It is important to consider the opinion of patients about improving their quality of life when assessing the efficacy of treatment.	9 (1)	100.00
50. It is important to consider the opinion of patients on the evolution of their symptoms when assessing the efficacy of treatment.	9 (1)	100.00
51. The routine use in clinical practice of symptom monitoring tools that include patient feedback may be good for anticipating the implementation of therapeutic actions that prevent the onset of severe symptoms.	8 (2)	91.49
52. The routine use in clinical practice of symptom monitoring tools that include patient feedback may ontribute to improving their quality of life.	9 (1)	93.61
53. Quality of life is a key element to be prioritized in the comprehensive approach to oncology patients with OIC.	9 (1)	100.00

*IQR* interquartile range, *OIC* opioid-induced constipation

### Topic 1. Diagnosis of OIC

The panel agreed all items proposed about the diagnosis of OIC in cancer patients, all of them with more than 70% of agreement (Table 1). Two items were agreed by all experts: the importance of awareness that OIC can be developed throughout opioid treatment and the recommendation of a proactive discussion between HCP and patients. Other relevant agreements included the use of Rome IV criteria to improve the differential diagnosis of OIC (82.97%), and the use of simple questionnaires with practical and measurable scales (87.24%). Of note, many panellists agreed that OIC is often not diagnosed correctly in cancer patients (85.10%). A complete clinical evaluation was recommended to rule out any other cause of constipation (metabolic, organic, pharmacologic, etc.) (95.75%).

### Topic 2. Treatment of OIC

Most of the items regarding OIC treatment in cancer patients reached consensus, all of them in agreement (Table 2). Prevention and early treatment were recommended to anticipate the development of OIC (97.87%). Once OIC is established, its management becomes more complicated (95.74%). Experts agreed that OIC treatment should remain for the duration of opioid treatment (91.48%).

OIC treatment should be kept as simple as possible, using an easy and convenient drug administration with a single daily dose (97.87%). These aspects will increase patient’ satisfaction (91.49%), which will help to improve the efficacy of OIC therapy (97.87%). The panel agreed that a good OIC therapeutic strategy requires the individualization of the treatment, adapting it to the needs of each patient. Furthermore, the panel broadly agreed that poor control of OIC can increase the number of medical visits, emergency visits, and healthcare costs (> 91.00%).

When OIC and functional constipation coexist in the same patient, a comprehensive approach of the situation is needed, treating specifically both types of constipation (91.49%).

The panel agreed that OIC needs a specific approach targeting the underlying cause (95.75%). Hygienic-dietary recommendations and laxatives, although necessary, are not effective enough (> 78.00%). Most of the experts agreed that oral PAMORAs are good therapeutic alternatives for the treatment of OIC in cancer patients (97.87%).

After laxative failure, panellist recommended to treat OIC using oral PAMORAs together with the laxative, maintaining the laxative doses as prescribed (80.00%). Osmotic laxatives were preferred for an adjuvant therapy with oral PAMORA (72.33%). In any case, panellists agreed that occasional use of enemas or opioid rotation should be considered when combined therapy with oral PAMORA and laxative was unsuccessful (> 72%).

### Topic 3. Quality of life of cancer patients with OIC

The panel agreed (97.9%) that OIC affects negatively the quality of life of cancer patient. According to all experts, it is important to consider patient’s opinion about the evolution of their OIC symptoms and quality of life when assessing the efficacy of OIC treatment. In addition, all experts believed that quality of life is a key element to be prioritized in the comprehensive approach of OIC in cancer patients. Most of the panellists agreed that regular assessment of patient-reported outcomes for symptom monitoring during routine clinical practice was a good option that may help to provide early treatment to patients’ symptoms preventing adverse consequences (91.49%), and may contribute to improving their quality of life (93.61%) (Table 3).

## Discussion

Despite the growing recognition of the burden of OIC in cancer patients leading to the development of new drugs targeting the underlying cause, few specific clinical guidelines for the management of OIC have been published [11, 13, 20, 24]. The present Delphi consensus was intended to be a useful tool to provide practical recommendations based on the experience of different Spanish physicians experts on opioid use and OIC.

OIC is a frequent problem among patients with cancer, and often underdiagnosed [14], as the panellist agreed. To identify effectively cancer patients with OIC, symptoms had to be assessed regularly since they can be present at the time of opioid prescription and throughout the treatment. It is recommendable to use simple, not time consuming, measurable assessment tools in clinical practice, like the Bowel Function Index (BFI), in line with previous recommendations [12, 14, 25, 26]. However, the BFI only collect patient-reported outcomes but not objective symptoms. Therefore, a more comprehensive tool based on Rome IV criteria and definition of OIC need to be developed.

For a successful management of OIC, functional constipation should be identified and treated before opioid treatment is initiated to avoid further complications. Previous study, observed that 71% of patient with constipation prior to opioid treatment had experienced exacerbation of symptoms with opioids [27].

The panel agreed OIC need specific and targeted management due to the specific pathophysiological features. For that reason, the first step is to be aware that OIC can be present at any time during opioid treatment and therefore OIC treatment should remain throughout opioid use. Prophylaxis, as well as early treatment, was considered essential to avoid further adverse events and complications. However, experts observed that despite recommendations [28], many patients on opioid treatment do not receive laxative prophylactic treatment, possibly because prevention in clinical practice is often merely informative.

Regarding conventional OIC therapy, the panel considered that addressing life-style aspects is important due to the multifactorial origin of constipation in cancer patients. However, changing life-style only do not alleviate OIC symptoms. Similarly, many times laxatives have shown poor efficacy [10, 16, 28, 29]. New therapeutic alternatives take on special value in this patient group. The new agents not only target the underlying cause of the problem, but also provide solution where there was none, introducing a new paradigm in the OIC treatment scheme [12, 14, 21, 25].

Panel agreed management of OIC should be tailored based on individual patient needs. Based on clinical practice, osmotic laxatives were barely considered first therapeutic option for OIC in cancer patients. Osmotic laxatives have been strongly endorsed for their efficacy in improving stool frequency and consistency in patients with chronic constipation [12, 30]. Furthermore, for the treatment of functional constipation osmotic laxatives have been recommended for hard stools, whereas stimulant laxatives are recommended for soft stools [30, 31]. However, these recommendations address other causes of constipation but not the problem of OIC, and to date, there is no enough evidence which suggest that one laxative is better than others [12, 32]. Therefore, the experts concluded that although osmotic laxatives could be more frequently used, given the paucity of the evidence there are insufficient data to make a general recommendation of one laxative over the other for the treatment of OIC in cancer patients. Clinicians should select laxatives based on the individual patient symptoms, needs, and performance status [12, 30, 31].

Oral PAMORAs were considered good therapeutic option for the treatment of OIC in cancer patient. According to latest publications, PAMORAs have been recommended for OIC when laxatives results in incomplete relieve of symptoms [12, 21]. Successful management of OIC requires a complete individual clinical evaluation being critical to establish the cause of constipation. A recent European expert consensus about OIC management has suggested to start treatment with an opioid antagonist if constipation was considered to be secondary to opioid therapy [14].

Moreover, cancer patients often suffer with mix aetiology constipation, and a comprehensive management should be installed. In this study, panel recommended co-prescription of laxatives with PAMORA in patients experiencing multifactorial constipation. Yet appropriate therapeutic scheme: laxative dose, laxative type, etc. need to be validated and requires further investigation. Definition of treatment failure is crucial for the success of OIC management, however it has been defined variably in the literature [12, 21, 26, 30]. Nonetheless, the panel recommended that treatment efficacy should be assessed as soon as possible and preferable within a week.

Finally, the panel evaluated the burden of OIC in the quality of life of cancer patients. According with previous data [9, 10, 33], panel agreed OIC negatively impact on the quality of life of cancer patients. Poorly controlled symptoms can result in increasing emergency department visits, unplanned hospitalizations, uncontrolled pain, delays in treatment, and lack of adherence and persistence with an effective treatment course. Patient perception of the burden of the problem, quality of life, and treatment has also a great impact on the success of the treatment and symptoms relief, therefore it should not be undervalued. Many clinicians are aware that improving cancer outcomes requires a focus not only on the main disease but also on patient illness experience and symptoms and their impact on the quality of life and their families. Systematic monitoring of patient-reported outcomes, including minor symptoms and patient experience, is now an essential component of cancer care [34–36].

The limitations of this study are similar to those of others with a comparable design. The promotor has not been involved in the development of the study, so a possible influence in the consensus has been minimized. One of the main strengths of this study is the participation of different kinds of physicians experts in OIC with high clinical experience. Far from being a disadvantage, this approach enriched and strengthened the consensus, since each item is evaluated from different points of view. In addition, the high degree of consensus reached give the study great validity of its results.

## Conclusion

Despite the improved knowledge of OIC physiopathology and management, standardized specific recommendations need to be developed to minimize the distress of OIC and to improve the quality of life of cancer patient. This modified Delphi consensus intended to provide with expert recommendations for the management of OIC in cancer patients, based on expert clinical practice. The main conclusions are as follow:

- Successful management of OIC requires increasing awareness of the impact of OIC in cancer patients among clinicians and patients.
- Higher proactivity to discuss OIC symptoms in clinical practice is recommended.
- The adoption of standardized tools to assess OIC symptoms (objective and subjective) in clinical routine practice is encouraged.
- OIC treatment should remain throughout opioid treatment and precise individualization based on patient symptoms, clinical status and preference.
- Oral PAMORAs are good alternatives for the treatment of OIC in cancer patients.
- Further research is needed to clarify the specific role of laxatives and oral PAMORAs in a comprehensive step-wise therapeutic strategy addressing the complexity of OIC in cancer patients.

## Supplementary Information

**Additional file 1.** Supplementary Table. Resume of the statements.

## Data Availability

All the datasets used and/or analysed during the current study available from the corresponding author on reasonable request. Supplementary information files are included in this published article.

## Abbreviations

OIC: Opioid-induced constipation
BFI: Bowel Function Index
HCP: Health care providers
PAMORAs: Peripherally Acting μ-Opioid Receptor Agonists
