# Supplementary material for: Delphi consensus on strategies in the management of opioid-induced constipation in cancer patients
Source: BMC Palliat Care. 2021 Jan 2;20:1. doi: 10.1186/s12904-020-00693-z (PMC7778791; doi:10.1186/s12904-020-00693-z)
Supplement: Supplementary file 1 — Additional file 1. Supplementary Table. Resume of the statements. [file 12904_2020_693_MOESM1_ESM.docx]

| ***Topic 1. Diagnosis of OIC recommendations and important facts*** |
| --- |
| The importance of awareness that OIC can be developed throughout opioid treatment. |
| Proactive discussion between HCP and patients. |
| Use of Rome IV criteria to improve the differential diagnosis of OIC. |
| Use of simple questionnaires with practical and measurable scales. |
| OIC is often not diagnosed correctly in cancer patients. |
| A complete clinical evaluation to rule out any other cause of constipation (metabolic, organic, pharmacologic, etc.). |

**Supplementary Table 1**. Resume of the statements

| ***Topic 2. Treatment of OIC recommendations and important facts*** |
| --- |
| Prevention and early treatment to anticipate the development of OIC. |
| Once OIC is established, its management becomes more complicated. |
| OIC treatment should remain for the duration of opioid treatment. |
| OIC treatment should be kept as simple as possible, using an easy and convenient drug administration with a single daily dose. These aspects will increase patient’s satisfaction and adherence of treatment, which will help to improve the efficacy of OIC therapy. |
| A good OIC therapeutic strategy requires the individualization of the treatment, adapting it to the needs of each patient. |
| A poor control of OIC can increase the number of medical visits, emergency visits, and healthcare costs. |
| Functional constipation should be treated before using opioids. |
| When OIC and functional constipation coexist in the same patient, a comprehensive approach of the situation is needed, treating specifically both types of constipation. |
| Laxatives are considered the first option to treat functional constipation in cancer patients who do not take opioids, maintaining the laxative dose when initiating opioid treatment. |
| OIC needs a specific approach targeting the underlying cause. |
| Hygienic-dietary recommendations and laxatives, although necessary, are not effective enough. |
| Oral PAMORAs are good therapeutic alternatives for the treatment of OIC in cancer patients. |
| After laxative failure, is recommended to treat OIC using oral PAMORAs together with the laxative, maintaining the laxative doses as prescribed. |
| Osmotic laxatives are preferred for an adjuvant therapy with oral PAMORA. |
| In any case, occasional use of enemas or opioid rotation should be considered when combined therapy with oral PAMORA and laxative was unsuccessful. |

| ***Topic 3. Quality of life of cancer patients with OIC recommendations and important facts*** |
| --- |
| OIC affects negatively the quality of life of cancer patient. |
| It is important to consider patient’s opinion about the evolution of their OIC symptoms and quality of life when assessing the efficacy of OIC treatment. |
| Quality of life is a key element to be prioritized in the comprehensive approach of OIC in cancer patients. |
| Regular assessment of patient-reported outcomes for symptom monitoring during routine clinical practice is a good option that may help to provide early treatment to patients’ symptoms preventing adverse consequences and may contribute to improving their quality of life. |
